# The tumor-associated YB-1 protein: new player in the circadian control of cell proliferation

**DOI:** 10.18632/oncotarget.14051

**Published:** 2016-12-20

**Authors:** Cristina Pagano, Orsola di Martino, Gennaro Ruggiero, Andrea Maria Guarino, Nathalie Mueller, Rima Siauciunaite, Markus Reischl, Nicholas Simon Foulkes, Daniela Vallone, Viola Calabrò

**Affiliations:** ^1^ Institute of Toxicology and Genetics (ITG) Karlsruhe Institute of Technology, Hermann-von-Helmholtz-Platz 1, 76344 Eggenstein-Leopoldshafen, Germany; ^2^ Department of Biology, University of Naples “Federico II”, 80126 Naples, Italy; ^3^ Institute for Applied Computer Science (IAI) Karlsruhe Institute of Technology, Hermann-von-Helmholtz-Platz 1, 76344 Eggenstein-Leopoldshafen, Germany

**Keywords:** circadian clock, cell proliferation, cell cycle, Y-box binding protein, SUMOylation

## Abstract

Correct spatial and temporal control of cell proliferation is of fundamental importance for tissue homeostasis. Its deregulation has been associated with several pathological conditions. In common with almost every aspect of plant and animal biology, cell proliferation is dominated by day-night rhythms generated by the circadian clock. However, our understanding of the crosstalk between the core clock and cell cycle control mechanisms remains incomplete. In this study, using zebrafish as a vertebrate model system, we show that the nuclear localization of the Y-box binding protein 1 (YB-1), a regulator of cyclin expression and a hallmark of certain cancers, is robustly regulated by the circadian clock. We implicate clock-controlled changes in YB-1 SUMOylation as one of the mechanisms regulating its periodic nuclear entry at the beginning of the light phase. Furthermore, we demonstrate that YB-1 nuclear protein is able to downregulate cyclin A2 mRNA expression in zebrafish via its direct interaction with the cyclin A2 promoter. Thus, by acting as a direct target of cyclic posttranslational regulatory mechanisms, YB-1 serves as one bridge between the circadian clock and its cell cycle control.

## INTRODUCTION

The circadian clock generates day-night rhythms in most aspects of physiology and behaviour [1]. Central to the circadian timing system is a pacemaker that oscillates with a period of circa 24 hours and is reset on a daily basis by environmental signals such as light, food or temperature, via input pathways. In turn, the master clock regulates physiology and behaviour via clock output pathways [1, 2].

At the core of the circadian clock mechanism is a transcription-translation feedback loop, which requires approximately 24 hours to complete one cycle. In vertebrates, the transcription factors CLOCK and BMAL activate expression of *Period* (*Per*) and *Cryptochrome* (*Cry*) genes, via binding to E-box enhancers in their promoters. Per and Cry in turn inhibit their own transcription by blocking CLOCK/BMAL driven transcriptional activation [3]. Additional transcriptional regulatory loops stabilize the entire timing mechanism [4]. Furthermore, cyclic post translational modifications, protein turnover and changes in the sub-cellular localization of key clock components all combine to reinforce the core clock mechanism's timing function [5–7].

One key mechanism regulated by the circadian clock is the timing of cell proliferation and circadian clock disruption has been associated with several pathological conditions [8–10]. Clock components have been implicated in controlling the expression of cell cycle regulatory genes in order to “gate” critical cell cycle steps to times of day when there is a reduced exposure to damaging ultraviolet light. Thus, the expression of several mammalian cell-cycle control genes such as *Wee-1* and *p21* has been found to be regulated in a circadian manner [11–13]. In addition, it has been shown that the clock has an impact on the cell cycle also through direct protein-protein interactions [14]. Other clock-controlled cell cycle regulators include *c-Myc*, *Mdm2*, the D1, A and B cyclins and the tumor suppressor *p53* [10]. There is also evidence that DNA damage can phase advance circadian rhythms via its effect on progression of the cell cycle [15]. Thus, the cell cycle can in turn also control the circadian clock. Therefore, understanding how these two key cellular oscillators, the clock and cell cycle, are inter-connected at the molecular level has become a major goal in the last few years.

The zebrafish (*Danio rerio*) has been established as one of the vertebrate model systems for studying the interplay between cell proliferation and the circadian timing system. In zebrafish, key cell cycle regulators, notably cyclins, are clock regulated and the circadian clock generates daily rhythms of S, as well as M phase by a cell-autonomous mechanism [16–19]. Most vertebrate tissues contain independent “peripheral” circadian clocks. However, while in mammals light entrainment of peripheral clocks occurs indirectly via the retina and the central clock of the suprachiasmatic nucleus (SCN) [20], zebrafish peripheral clocks are entrained by direct exposure to light [21]. In turn, direct exposure to light-dark cycles result in high amplitude rhythms of cell proliferation in zebrafish cells and tissues.

One of the key regulators of cyclin expression during cell cycle progression [22] is the Y-box binding protein 1 (YB-1, YBX-1). High YB-1 levels are typical of regenerating and proliferating cells and its abnormally elevated expression is a hallmark of cancer progression [23]. YB-1 is a DNA/RNA binding protein involved in almost all DNA and mRNA-dependent process, including DNA replication and repair, transcription, pre-mRNA splicing and translation. This protein is composed of three domains: a protein-protein interaction A/P rich N-terminal domain; a five stranded β-barrel CSD (Cold shock domain) [24] and a large C-terminal domain involved in controlling the subcellular localization of the protein [22, 25–27]. Although most YB-1 is located in the cytoplasm, recent evidence has shown that YB-1 can shuttle between the nucleus and the cytoplasm [28]. In the nucleus, YB-1 orchestrates expression of proliferation-related genes, whereas in the cytoplasm it associates with mRNA and directs translation. YB-1 relocalization from the cytoplasm to the nucleus at the G1/S phase transition has been associated with its function of regulating cyclin gene expression [22, 29, 30]. In this regard, in the nucleus YB-1 protein has been demonstrated to bind DNA as well as RNA, in a sequence-specific fashion to an inverted CCAAT box sequence (5′- CTGATTGGC/TC/TAA - 3′), termed the Y-box [31].

Here, using zebrafish as a model, we implicate YB-1 as one of the links between the circadian clock and the control of cell cycle progression. We reveal a circadian clock control of YB-1 sub-cellular nuclear localization involving cyclic changes in SUMOylation and that this regulation is, at least in part, responsible for circadian cell cycle regulation.

## RESULTS

### Clock regulation of nuclear zfYB-1

**Figure 1:**
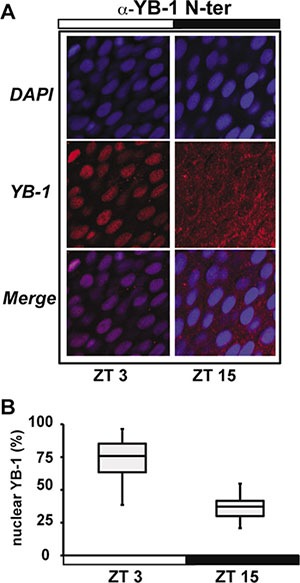
zfYB-1 cellular localization in zebrafish caudal fins (**A**) Immunofluorescence analysis of zfYB-1 protein in the caudal fin at ZT3 (light phase) and ZT15 (dark phase) using α-YB-1 N-ter antibody. Panels also show DAPI staining and Merge, which combines both the DAPI and YB-1 signals. White and black bars indicate the corresponding lighting conditions. (**B**) Quantification of the panel A.

**Figure 2:**
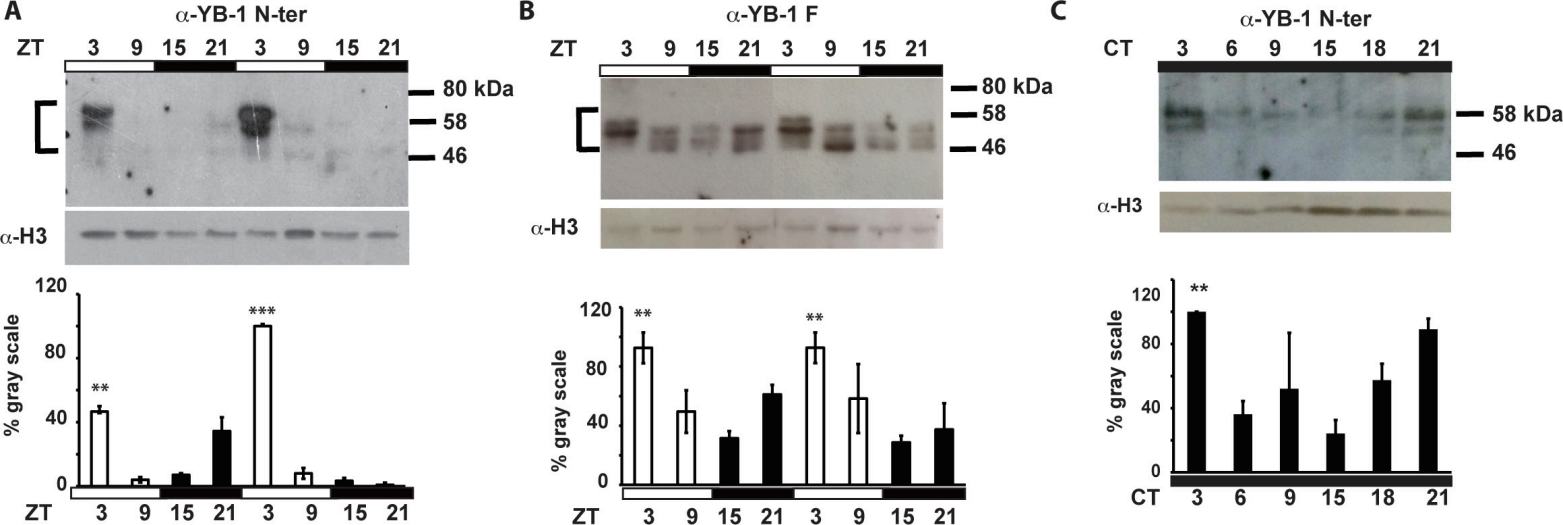
zfYB-1 protein expression in caudal fins Western blot analysis and quantification of zfYB-1 levels in nuclear extracts under LD cycles (**A**, **B**) or during the second day in constant darkness, DD (**C**). The specific antibody used to detect YB-1 protein is indicated above each panel. An α-H3 antibody was used to provide a loading control. Vertical “brackets” on the left side of the panels A and B demarcate the YB-1 immunoreactive bands that were quantified using Image Lab™ Software. Quantification was expressed as % of gray scale relative to the highest peak of expression and is plotted on the Y-axes. Statistical significance between peak and trough points is indicated by asterisks where *p* < 0.05, *p* < 0.001 and *p* < 0.0001 are represented by *, ** and *** respectively. Results of the CircWave analysis are represented in Supplementary Table S1.

We have previously shown that the transcription of cell cycle control genes, including *zfcyclin A2* and *zfcyclin B1*, is regulated by the circadian clock in the adult zebrafish caudal fin [16]. Given the central role played by YB-1 in the control of the cell cycle, we wished to investigate its involvement in circadian clock-regulated cell proliferation in zebrafish. It is well documented that the regulation of gene expression exerted by the human YB-1 protein is mainly due to dynamic changes in its nuclear localization [22, 32]. Therefore, based on the high homology between human and zfYB-1 (Supplementary Figure S1), we performed immunohistochemical analysis of zebrafish caudal fins at two different times during the light-dark cycle (14:10) using three different antibodies raised against full length, the N-terminal and the C-terminal portions of the human YB-1 protein, named: α-YB-1 F, α-YB-1 N-ter and α-YB-1 C-ter, respectively (Figure 1 and Supplementary Figure S2A–S2C and Supplementary Table S4). With all three antibodies we observed an enriched nuclear localization at zeitgeber time (zt) 3 (day time) compared with zt 15 (night time), where zt 0 is defined as “lights on“ and zt 14 “lights off” (Figure 1A, 1B and Supplementary Figure S2B, S2C and Supplementary Table S1 for statistical analysis). This daily change in zfYB-1 nuclear localization, was confirmed by western blot analysis of fin nuclear extracts harvested at 6 hourly intervals during 2 Light/Dark (LD) cycles (Figure 2). A statistically significant sinusoidal rhythm in the nuclear expression of immunoreactive zfYB-1 with a peak at zt 3 and a trough at zt 15 was observed (Figure 2A, 2B and Supplementary Table S1 for statistical analysis).

Consistent with previous studies where multiple YB-1 immunoreactive bands were associated with complex post translational modifications [27, 31, 33], we also observed differences in the number and molecular weight of immunoreactive bands depending on the antibody used (ranging between 45 and 60 kDa). In addition, the oscillation of nuclear YB-1 expression persisted under constant conditions (constant darkness, DD) showing a circadian clock regulation rather than simply a light-dependent YB-1 rhythmicity (Figure 2C and Supplementary Figure S2D and Supplementary Table S1). Then, using a combination of approaches based on siRNA, recombinant protein and immunoprecipitation assays, we confirmed that the immunoreactive bands observed using the YB-1 antibodies, indeed correspond to zfYB-1 protein (Supplementary Figure S3A–S3D).

One frequently encountered feature of genes that serve to bridge the circadian clock with its output pathways is that their mRNA expression exhibits clock driven rhythms. We thus performed a high-resolution qRT-PCR analysis of *zfYb-1* mRNA levels in caudal fins prepared from zebrafish raised under LD cycles and then transferred to constant darkness (DD). At the RNA level, *zfYb-1* showed no significant circadian rhythm (Supplementary Figure S4A and Supplementary Table S1), unlike for *zfper1b* and *zfcyclin A2* which both show a predominant circadian clock regulation (Supplementary Figure S4B, S4C and Supplementary Table S1). However, basal *zfYb-1* mRNA levels did show a progressive increase after transfer to constant darkness implicating light in setting the levels *zfYb-1* mRNA expression (Supplementary Figure S4A and Supplementary Table S1).

### Cell autonomous regulation of nuclear zfYB-1 by the circadian clock

**Figure 3:**
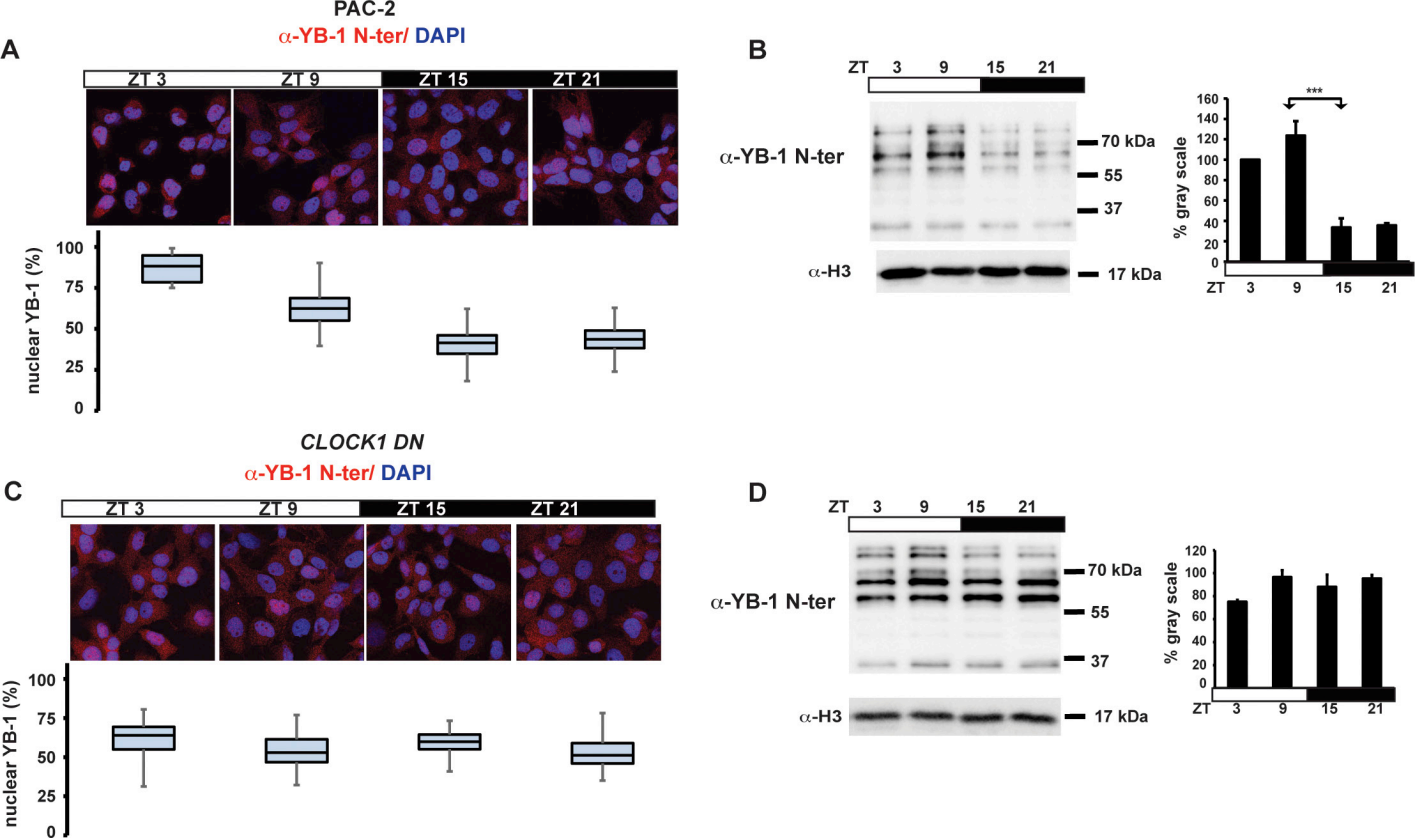
Nuclear zfYB-1 expression in PAC-2 and CLOCK1 DN cells (**A**, **C**) Immunofluorescence analysis and quantification of nuclear levels of immunoreactive zfYB-1 in PAC-2 (A) and CLOCK1 DN (C) cells using the α-YB-1 N-ter antibody. Cells maintained under an LD cycle were fixed at 4 time points (ZT). A Merge of the DAPI and YB-1 staining images are shown. (**B**, **D**) Western blot analysis (right) and quantification (left) of α-YB-1 levels in fin nuclear extracts in PAC-2 (B) and CLOCK1 DN (D) cells under LD conditions and normalized for H3 expression (α-H3). Quantification was expressed as % of gray scale relative to the highest peak time point (ZT 9) and is plotted on the y-axis. ZT times are plotted on the x-axis. Statistically significant differences between peak and trough points are indicated by asterisks (*). Results of the CircWave analysis are represented in Supplementary Table S1.

Consistent with a cell autonomous clock-regulation, immunohistochemical and western blot analysis also revealed a robust oscillation of zfYB-1 nuclear protein in zebrafish PAC-2 cells (Figure 3A, 3B and Supplementary Figure S5A, S5B and Supplementary Table S1), although compared with fins, levels remained elevated in the nuclei for a longer period (peak zt3-9). We next wished to test whether the observed 24 h rhythmicity of nuclear zfYB-1 was directly driven by the core circadian clock mechanism or merely reflected clock-dependent rhythmicity of cell cycle progression. With this goal, we explored the effect of genetic clock disruption or cell proliferation arrest upon zfYB-1 nuclear rhythmicity. Specifically, in the first approach we employed PAC-2 cells stably expressing a dominant negative form of the zfCLOCK1 protein, *CLOCK1 DN*, which has been show to disrupt core clock function (Supplementary Figure S5C–S5E) [34]. In *CLOCK1 DN* cells exposed to a LD cycle, we observed no significant rhythmicity in nuclear zfYB-1 protein (Figure 3C, 3D and Supplementary Table S1). In the second approach, we inhibited PAC-2 cell proliferation by serum deprivation at a high level of cell confluence. Under this condition, we observed rhythmicity of nuclear zfYB-1 levels under LD cycles, which also persisted on the second day following transfer to DD conditions (Supplementary Figure S5F and Supplementary Table S1). Together, these data confirm that rather than simply reflecting clock-dependent rhythmicity of cell cycle progression, zfYB-1 nuclear oscillation is a target of the CLOCK/BMAL-based core clock mechanism.

**Figure 4:**
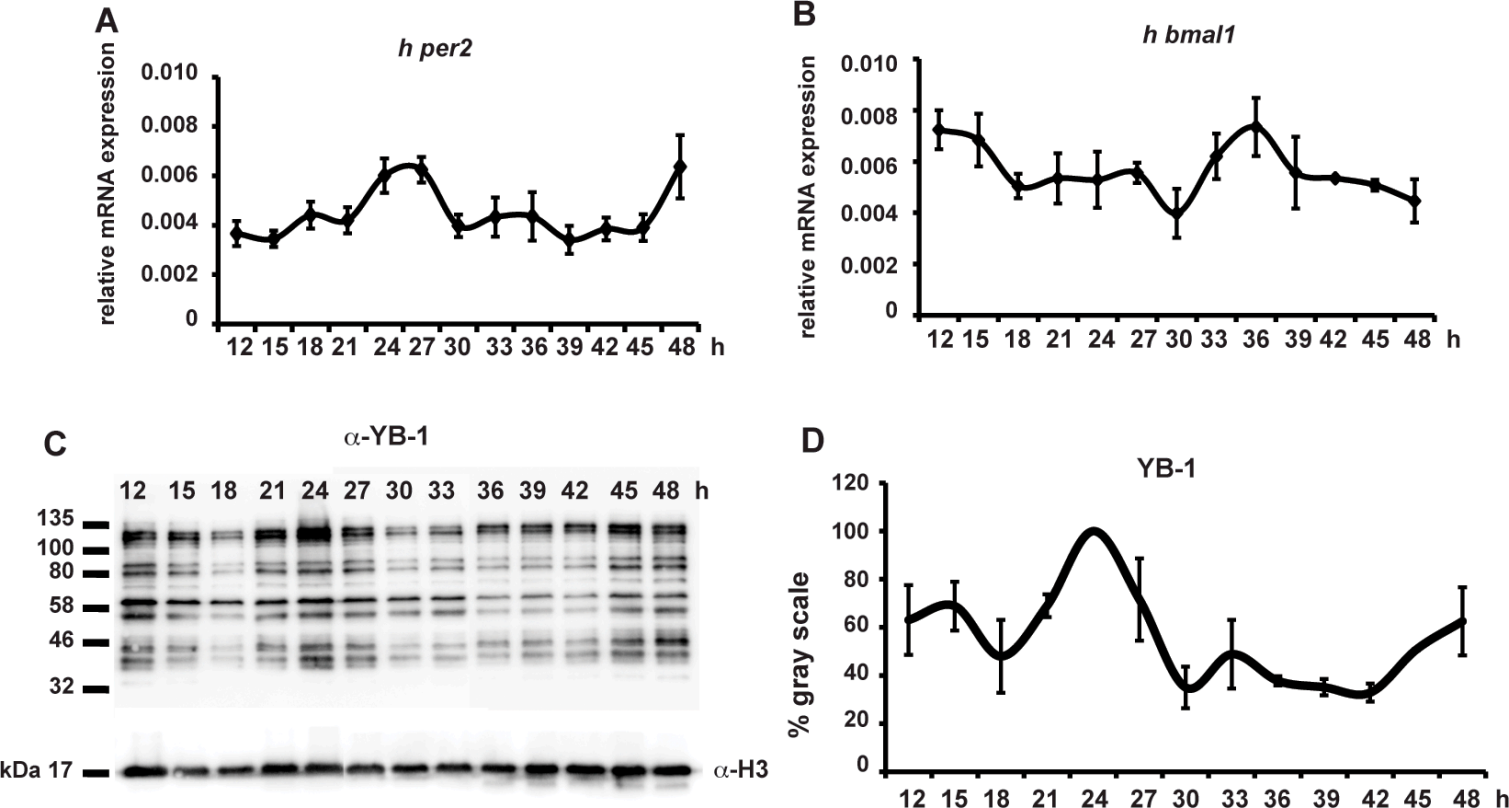
hYB-1 expression in clock synchronized HEK293 cells (**A**, **B**) Real time qRT-PCR analysis of *hper2* and *hbmal1* expression in HEK293 cells during 48 hours following transient treatment with 100 nM of Dexamethasone. Relative mRNA levels are plotted on the y-axis. Times after DEX treatment are plotted on the x-axis. Levels of *h-gapdh* mRNA were used for normalization. Results of the CircWave analysis are represented in Supplementary Table S1. (**C**) Western blot analysis using the α-YB-1 N-ter antibody in HEK293 cell nuclear extracts prepared after DEX treatment. H3 expression was used as a loading control. (**D**) Quantification analysis of panel (C). Quantification was expressed as % of gray scale relative to the highest peak time point (24 hrs) and plotted on the y-axis. Times after DEX treatment are plotted on the x-axis. The result of the CircWave analysis is represented in Supplementary Table S1.

Given the importance of YB-1 function in mammalian cells, we also wished to explore whether this circadian clock regulation of YB-1 nuclear localization is an evolutionary conserved mechanism. Thus, we entrained the circadian clock in human HEK 293 cells via transient treatment with dexamethasone and then assayed nuclear YB-1 levels during the subsequent 48 h (Figure 4). In treated cells, clock synchronization was confirmed by circadian, antiphase rhythms of mRNA expression in two core clock genes, *hper2* and *hbmal1* (Figure 4A, 4B and Supplementary Table S1). In these cells, we also observed a circa 24 h oscillation of nuclear YB-1 protein (Figure 4C, 4D and Supplementary Table S1). Therefore, all our results point to the cell-autonomous circadian clock finely tuning the nuclear localization of the zfYB-1 protein via an evolutionary conserved mechanism.

### YB-1 SUMOylation in PAC-2 cells

**Figure 5:**
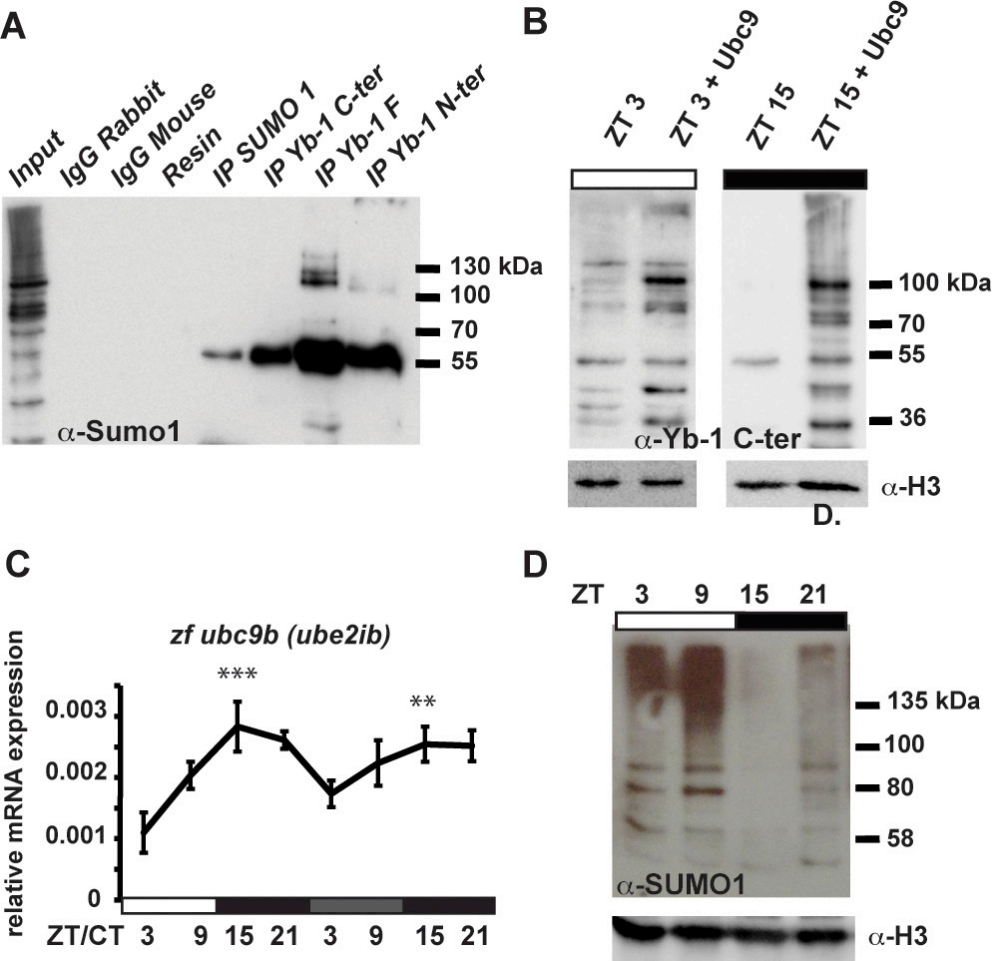
zf YB-1 SUMOylation (**A**) Western blot analysis with the α-SUMO-1 antibody of IPs prepared from total cell extracts using each of the three α-YB-1 antibodies. Input and negative controls are indicated. (**B**) Western blot analysis using the α-YB-1 C-ter antibody in PAC-2 cell nuclear extracts prepared at ZT3 and ZT15 under an LD cycle in the absence or presence of over-expressed SUMO-conjugating enzyme UBC9 (+Ubc9). H3 expression was used as a loading control. (**C**) Real time qRT-PCR analysis of *zfubc9b* expression during exposure to a LD cycle followed by transfer to DD. Relative mRNA levels are plotted on the y-axis. ZT and CT times are plotted on the x-axis. *β-actin* mRNA was used for normalization. Statistical significance between peak and trough values in LD and in DD are indicated by asterisks. (**D**) Western blot analysis of SUMO-1 immunoreactive proteins in nuclear extracts prepared from cells under LD cycles. H3 expression was used as a loading control. Results of the CircWave analysis are represented in Supplementary Table S1.

How does the clock regulate zfYB-1 nuclear localization? One mechanism that has been implicated in clock-directed shuttling of proteins between the nucleus and cytoplasm is SUMOylation [5, 35]. SUMOylation has been documented to time the nuclear entry of the BMAL1 clock protein. Using GPS-SUMO 1.0 software we identified three potential conserved SUMOylation target sites in the zfYB-1 protein, one of which is a canonical inverted site (DSKA in position 287–290) and the other two are non-canonical sites (TKED 60–63 and EKRE 151–154) (Supplementary Figure S1). Extracts from PAC-2 cells immunoprecipitated (IP) at zt 3 with each of the three YB-1 antibodies independently, were subjected to immunoblotting for SUMO-1. Our results showed that the IP proteins from all three YB-1 antibodies were also recognized by the SUMO-1 antibody, consistent with the presence of SUMOylated YB-1 in zebrafish (Figure 5A). Furthermore, overexpression of the SUMO-conjugating enzyme UBC9 resulted in an increase in the overall quantity of zfYB-1 nuclear protein at zt 3 and also an increase in molecular weight observed at zt 15 (Figure 5B and Supplementary Figure S5B for comparison). To explore how the clock could regulate the YB-1 SUMOylation events, we next investigated the possibility that the expression of a subset of genes involved in SUMOylation could also be clock regulated. Real time qRT-PCR analysis in PAC-2 cells exposed to a LD cycle followed by DD conditions revealed daily rhythmic expression of *zfubc9* (*zfube2ia*, *zfube2ib*), *zfsumo1*, *zfsae1* (SUMO1 activating enzyme subunit 1) and *zfpias1* (E3 SUMO-protein ligase PIAS1) with a peak around the light–dark transition, which also persisted in most cases under DD conditions (Figure 5C, Supplementary Figure S6 and Supplementary Table S1). In addition, western blot analysis of PAC-2 cells for proteins covalently linked with SUMO-1 groups showed a day-night rhythm in the global levels of nuclear SUMOylated protein that is consistent with the timing of zfYB-1 nuclear localization (Figure 5D). Therefore, together these data point to a more general control of protein SUMOylation by the circadian clock rather than only YB-1.

### Inhibition of *zfcyclin* A2 expression by zfYB-1

Previous reports have demonstrated that YB-1 plays a key role in regulating cell proliferation. In the nucleus, YB-1 orchestrates expression of many proliferation-related genes. Therefore, we wished to explore the functional consequences of daily changes in nuclear YB-1 levels in zebrafish cells. FACS analysis of PAC-2 cells over expressing zfYB-1 and exposed to a LD cycle assayed at 6 hourly intervals during 24 h, showed a loss of cell cycle rhythmicity with an elevation in the proportion of cells in S and G2/M phase and a reduction in G0/G1 cells (Supplementary Figure S7A). Therefore, YB-1 has an impact on the dynamics of cell cycle progression in zebrafish cells.

**Figure 6:**
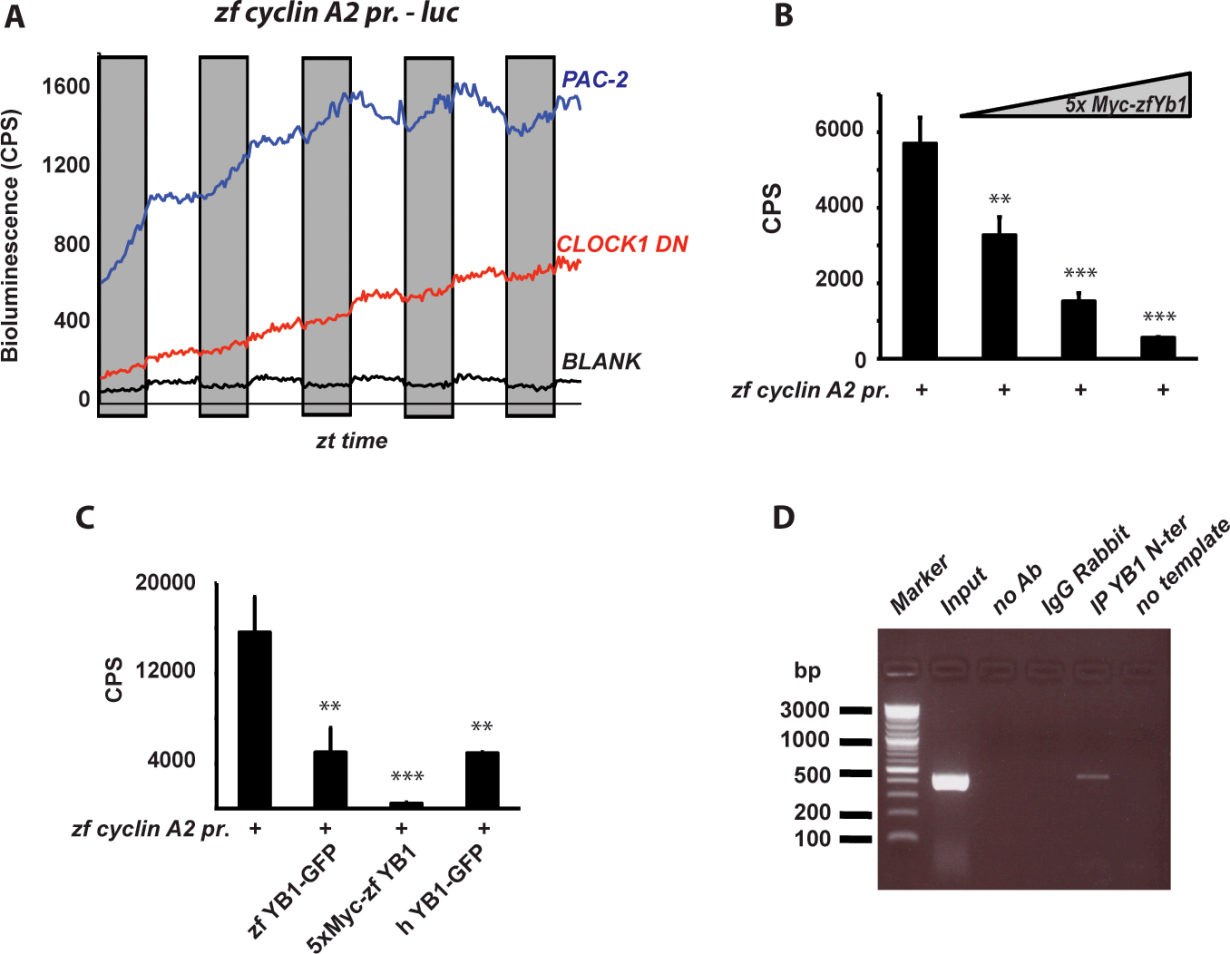
Regulation of zf Cyclin A2 expression by zfYB-1 (**A**) Graphical representation of the real time bioluminescence assay results from PAC-2 (blue trace) and CLOCK1 DN cells (red trace) transfected with the *cyclin A2 pr*. luciferase reporter. The black trace (BLANK) represents the luminescence background of the cell plate. The bioluminescence in counts per second (CPS) is plotted on the y-axis and the ZT times on the x-axis. Results are plotted as the mean of 4 independent transfections. Gray bars indicate the corresponding dark periods. (**B**–**C**) *In vitro* luciferase assays in cells co-transfected with the *cyclin A2 –pr*. reporter and (B) 25, 50 and 100 ng of the 5xMyc-zfYB-1 expression vector or (C) with 50 ng of the zfYB-1-GFP, 5xMyc-zfYB-1 or the hYB-1-GFP expression vectors. Data are represented as means of triplicate samples +/– SD. At least three independent experiments were analyzed. Data were standardized for transfection efficiency using a β-galactosidase assay. Statistical significance is indicated above the graph by asterisks (*). (**D**) Agarose gel showing the 447 bp DNA promoter product amplified from ChIP assays using primers shown in Supplementary Table S2. Input and negative controls are indicated.

It has been previously shown that the mRNA expression of cyclins in zebrafish tissues is clock regulated [16, 18, 19]. Consistently, we confirmed the clock regulation of *zfcyclin A2* mRNA expression in PAC-2 cells (Supplementary Figure S7B). To address the role of zfYB-1 in cell cycle control we analyzed the contribution of zfYB-1 to the regulation of *zfcyclin A2* mRNA expression. Thus, we cloned a 447 bp fragment of the *zfcyclin A2* promoter including its 5′-UTR region, upstream of a luciferase reporter (*zf cyclin A2 prom*). This region contains 4 putative YB-1 binding sites, 3 of them in the promoter and 1 in the 5′UTR region (Supplementary Figure S7C). Expression of this reporter construct in cells exposed to LD cycles increases progressively over the course of the assay but at a significantly higher rate during the dark phase (Figure 6A and Supplementary Figure S7D and Supplementary Table S1). Consistent with this reflecting regulation by the circadian clock, in transfected *CLOCK1 DN* cells this rhythmicity was not observed (Figure 6A and Supplementary Table S1). The higher cyclin A2 reporter gene expression during the dark period correlates with lower nuclear levels of zfYB-1 protein (see Figure 3A, 3B). In support of zfYB-1 playing a negative regulatory role, we observed a dose dependent inhibition of zf*cyclin A2* promoter expression after co-transfection with the expression constructs for zebrafish YB-1 (5′Myc-tag or 3′GFP-tag zfYB-1) and human YB-1 (3′GFP-tag hYB-1) (Figure 6B, 6C and Supplementary Figure S3D for controls) in PAC-2 cells. Finally, using a chromatin immunoprecipitation (ChIP) assay, we revealed a direct interaction between zfYB-1 and the *zfcyclin A2* promoter (Figure 6D and Supplementary Figure S7E). Therefore, together our results are consistent with a role of zfYB-1 in the circadian clock control of *cyclin A2* expression.

## DISCUSSION

Circadian rhythmicity is one of the defining features of normal cell cycle progression in many highly proliferative tissues *in vivo*. Furthermore, frequent disruption of the circadian clock has been linked with an increased risk of many pathologies including cancer. For this reason, a major goal is to understand the links between the core clock mechanism and key cell cycle control systems. Here, using the zebrafish as a model, we have implicated YB-1 as one such element. The sub-cellular localization of this protein changes significantly with the time of day and high nuclear levels are associated with the early day period. These dynamic changes in YB-1 location are driven by the cell autonomous circadian clock mechanism and involve SUMOylation, a post-translational modification already implicated in core clock function [5]. We show that in the nucleus, YB-1 physically associates with the cyclin A2 gene 5′ regulatory region and down-regulates its expression.

Previous findings have pointed to considerable complexity in YB-1 function consistent with a central role in the regulation of cell proliferation. However, the majority of these studies have focused on the role of YB-1 in tumorigenesis with comparatively little attention being applied to its normal physiological function. Although most YB-1 is located in the cytoplasm, recent evidence has shown that YB-1 can shuttle between the nucleus and the cytoplasm [28]. In the nucleus, YB-1 orchestrates expression of proliferation-related genes, whereas in the cytoplasm it associates with mRNA and regulates translation. Here we present data linking high nuclear levels of YB-1 with repression of *cyclin A2* expression. Cyclin A2 is synthesized at the onset of S-phase and then localizes to the nucleus where it is implicated in the initiation and progression of DNA synthesis. Our findings are consistent with previous results implicating YB-1 as a cell cycle stage-specific transcription factor [22] present in the nucleus at the boundary between G1 and S phase of the cell cycle. However, the precise manner whereby YB-1 regulates cyclin gene expression remains uncertain. Nevertheless, it is tempting to speculate that the clock control of YB-1 might serve as part of a more general mechanism to restrict DNA replication to a specific temporal window to minimize the impact of UV light-induced DNA damage. However, given the multiple cellular mechanisms which involve YB-1, this clock related function is almost certainly not the only role played by the YB-1 protein.

Many lines of evidence also point to tight control of the timing of DNA repair processes during the day-night cycle. Thus, robust circadian rhythmicity of DNA replication which is anti-phase to that of DNA repair has been reported [36]. Furthermore, in zebrafish the expression of many genes involved in DNA repair is strongly induced by light, thus leading to an increase in DNA repair capacity during periods of maximum sunlight-induced DNA damage. In addition, DNA damage itself has been shown to constitute a zeitgeber for the circadian clock [15]. Thus, circadian clock gating of the cell cycle may also optimally time DNA damage repair. In this respect, it is interesting to note that nuclear YB-1 has been demonstrated to have anti-apoptotic properties based on its ability to coordinate DNA excision repair function and protect cells against damaging agents [37]. Furthermore, various forms of stress, including oxidative, genotoxic stress and hyperthermia induce nuclear translocation of YB-1 [31, 38] implicating YB-1 in stress and DNA damage responses.

Cell autonomous, circadian clock control of cellular processes frequently occurs at the level of transcription initiation. Circadian rhythms in the expression of many clock-controlled genes (ccgs) are directed by circadian E-box enhancer promoter elements that represent the regulatory targets of the core clock proteins CLOCK and BMAL1. However, a host of other proteins, including kinases and phosphatases [39], chromatin modifiers [40] and RNA-binding factors [41] in turn play essential roles in conferring robustness on the core circadian clock mechanism. YB-1 is constitutively expressed in proliferating tissues and performs a multitude of cellular functions, thus it is predictable that certain YB-1 functions are regulated by reversible post-translational modifications (PTM) rather than transcriptional mechanisms. In this regard, our results implicate SUMOylation in the clock-controlled translocation of YB-1 into the nucleus. Interestingly, circadian rhythmicity in the SUMOylation of the core clock protein, BMAL1, that is mediated by CLOCK, has been implicated in the regulation of nuclear entry [42]. Furthermore, our data point to general circadian rhythmicity in the mechanisms controlling SUMOylation. Thus, we speculate that YB-1 is the target of a cycling SUMOylation mechanism that relays timing information both within and outwards from the core clock mechanism.

Among the hallmarks of cancer, genome instability and mutations in cell cycle genes are recurring enabling factors [10]. Indeed mutations in cell cycle genes have been encountered in 90% of human cancers [43]. However, disruption of normal circadian clock function has also been linked with tumorigenesis [44]. At an epidemiological level, shift work, chronic jet lag and other environmental factors impacting on circadian clock function are also linked with increased cancer development and disrupted cell proliferation. Many human cancer cell lines that by definition exhibit abnormal cell cycle progression, also display abnormal circadian clock gene expression [9, 10]. Furthermore, mouse knock out lines affecting certain core clock genes show an increased susceptibility to tumor formation [45, 46]. While expression of YB-1 protein is frequently elevated in tumors, its precise role in tumorigenesis is still poorly understood. In human tumors, such as breast, lung and prostate cancer, elevated YB-1 protein levels and nuclear localization [47] appear to be prognostic, indicating poor clinical outcome. Furthermore, the accumulation of YB-1 protein in the nucleus is associated with increased cell survival and multidrug resistance [31, 48]. However, YB-1 over-expression has also been reported to block oncogenic transformation [49]. These apparently contradictory results may be the consequence of differential sub-cellular localization. Specifically, in the cytoplasm YB-1 may interfere with oncogenic transformation as a result of its function in translational control [25, 50]. YB-1 regulates the expression of several tumor-associated genes [51, 52]. As a result, elevated levels of YB-1 may play a role in facilitating tumor cell invasion and metastasis, as well as enhancing cell growth and resistance to chemotherapeutic agents. Thus, it is tempting to speculate that disruption of normal circadian rhythmicity of YB-1 function may serve as a key link with tumorogenesis.

## MATERIALS AND METHODS

### Fish care, treatment and ethical statements

Zebrafish (*D. rerio*) were maintained according to standard procedures [53] in a re-circulating water system at 28°C and under 14:10 light : dark cycles. For each experiment 6–12 months old zebrafish males or females were used. Under constant darkness, the fish were fed twice daily at random time points using automatic feeders. The caudal fins were amputated using razor blades following anesthesia with 0.02% w/v MS222 (3-aminobenzoate methanesulfonic acid, Sigma Aldrich). All zebrafish husbandry and experimental procedures were performed in accordance with the German animal protection standards (Animal Protection Law, BGBl. I, 1934 (2010)) and were approved by the Local Government of Baden-Wurttemberg, Karlsruhe, Germany (Az.: 35-9185.81/G-130/12 and 35-9185.81/G-131/16). General license for fish maintenance and breeding: Az.: 35-9185.64).

### Quantitative RT-PCRs

Total RNA was extracted with Trizol Reagent (Gibco, BRL) according to the manufacturer's instructions. Reverse transcription was performed using Superscript III RT (Invitrogen). A StepOnePlus Real-Time qRT-PCR System (Applied Biosystems) and SYBR Green I fluorescent dye (Promega) were used. Expression levels were normalized using *zf β-actin* and *h-gapdh* mRNA expression for zebrafish and human HEK 293 cells, respectively. The relative levels of mRNA were calculated using the 2^−ΔΔCT^ method. For each gene, primer sequences are presented in Supplementary Table S2.

### RNA interference

zfYB-1, NM_001126457, transient silencing was carried out in PAC-2 cells with increasing concentrations of IBONI YB-1 siRNA (RIBOXX GmbH, Germany) and FuGene HD reagent (Promega) according to the manufacturers’ recommendations. iBONi siRNA Negative Control was provided by Riboxx as a pool of 3 different siRNA. In Supplementary Table S3, all the siRNA sequences utilized are presented.

### Protein analysis

Nuclear and cytoplasmic protein extracts were obtained using the NP40 lysis buffer protocol as described elsewhere [54]. Total protein extracts were also prepared by directly adding 200 μl of 1X Laemmli buffer (6% SDS, 20% glycerol, 125 mM TrispH6.8, 0,01% bromophenol blue, 100 mM DTT) including 1× cocktails of protease and phosphatase inhibitors (Sigma Aldrich) to the fin tissue or the cells. Gel electrophoresis was performed in a SDS polyacrylamide gel and proteins transferred to a Hybond-P membrane (Millipore). Binding of each antibody was visualized using the ECL detection system (Biorad). All images were acquired and analyzed with Image Lab™ Software (Bio Rad, USA) or with X-Ray films (Kodak). Then, images were quantified using the Scion Image software (NIH, http://rsb.info.nih.gov/nih-image/). A list of the primary and secondary antibodies used is shown in Supplementary Table S4. YB-1 immunofluorescence analysis of fins was performed after overnight fixation of fin-clips in Carnoy's solution (60% ethanol, 30% chloroform, 10% acetic acid) at 4°C followed by an incubation overnight in 100% Methanol and sequential rehydration steps in 100%, 66% and 33% methanol in PBTX (1XPBS, 0,3% Triton X100). Instead, adherent cells (1.2 × 10^5^) were fixed overnight at 4°C in 4% PFA in 1× PBS and then transferred to PBST (1X PBS plus 0,1% Tween 20). At this stage, fins and cells were pre-incubated in blocking solution (PBTX and PBST, respectively plus 1% BSA (GE healthcare) for 3 hrs. Then the primary antibody was added and the samples were incubated at 4°C overnight. After several washes, the samples were incubated at 4°C with the secondary fluorescent antibody (Supplementary Table S4) for 2 hours (cells) or overnight (fins). DAPI staining was used for visualization of nuclei. The samples were mounting on glass slides and photographed using a Confocal Microscope SPE (Leica) with a 40X oil immersion objective and the quantification of nuclear and cytoplasmic signals was calculated using Image Lab™ Software (Bio Rad, USA). Co-Immunoprecipitations (Co-IPs) were performed as previously described [32]. The antibody concentrations used in each IPs and for western blotting analysis were chosen according to the manufacturers’ recommendations.

### Cell cultures and clock entrainment

The zebrafish cell line PAC-2 [55] was propagated as previously described [56].

The zebrafish *CLOCK1 DN* cells were a kind gift of David Whitmore and were propagated as previously described [34]. The HEK293 (human embryonic kidney derived) cell line was cultured at 37°C with 5% CO_2_ in Dulbecco's Modified Eagle's Medium (DMEM) containing 10% heat inactivated fetal calf serum (FCS), 2 mM L-glutamine, 50 μg/ml penicillin and 50 μg/ml streptomycin.

The circadian clock in zebrafish cells was entrained by direct exposure of the cells to light–dark cycles at a constant temperature of 25°C using white light emitting diodes (LED, Kopa 440 nm-690 nm) adjusted to deliver a photon flux of 1.42 × 10^18^ ± 0,04 ×10^18^ photons/s/m^2^_·_

The clock entrainment in the HEK293 cells was performed by plating the cells at a concentration of 2.5 × 10^6^ in DMEM medium (Sigma-Aldrich) in the presence of 10% serum. At 80% confluence, the concentration of serum was reduced to 2%. After 24 h, the cultures were treated with 100 nM water-soluble dexamethasone (Sigma-Aldrich) to synchronize the cells (time = 0 h), and after 2 h of incubation the medium was replaced with DMEM culture medium containing 2% serum. All cultures were incubated at 37°C in a humidified 5% CO_2_ atmosphere until harvesting at specific time points after dexamethasone treatment (from 12 h to 48 h) according to the specific analysis.

### FACS analysis for flow cytometry

Cells were trypsinized, collected and washed two times in cold 1x PBS via centrifugation at 1200 rpm for 4 minutes at 4°C. Next, the cell pellets were resuspended in 0.5 ml of ice-cold 100% methanol and incubated on ice for 20 minutes. After centrifugation at 1200 rpm for 4 minutes at 4°C, the pellets were resuspended again in cold 1× PBS and incubated on ice for 30 minutes prior to centrifugation and final resuspension in 0.5 ml of 1× PBS in the presence of RNAase at a final concentration of 100 ug/ml. After 20 minutes of incubation in RNAase at room temperature, the samples were finally stained with 50 ug/ml of Propidium iodide (PI) (Sigma) for 30 minutes on ice and in darkness. Data acquisition and analysis of the cell cycle phases were performed using a BD Accury C6 cytometer and software from BD Biosciences. Each time point was analyzed in triplicate, 40.000 events were analyzed for each sample.

### Luciferase assays

Cell transfections were performed using FuGene HD (Promega) or ScreenFect (S-4001 InCella) reagents according to the manufacturers’ protocols. β-galactosidase expression was used to normalize for transfection efficiency using a standard protocol [57]. Luciferase activity was measured using the Luciferase Assay System kit (Promega) and a VICTOR Multilabel Plate Reader (Perkin Elmer). The real-time bioluminescence assays were performed and analyzed as described previously [56, 58]. Bioluminescence was assayed with a Topcount NXT automatic scintillation counter (Perkin Elmer). Data were imported into Microsoft Excel using the ‘‘Import and Analysis’’ macro (S. Kay, Scripps Research Institute).

### Constructs

The expression constructs 5xMyc-zfYB-1 and zfYB-1–GFP and the *zfcyclin* A2 promoter were cloned by PCR amplification of PAC-2 cell DNA using pfu polymerase (Promega) and then subcloned into the pCS2-MTK 5xMyc (Invitrogen), pcDNA6-V5/HisB GFP (ThermoFisher) and pGL3 basic-Luc (Promega) expression vectors (see Supplementary Table S5 for details). The hYB-1-GFP expression vector was previously described (24). The p3259 pCMV hUBC 9 mt HA was a gift from Peter Howley ADDGENE (14439) [59].

### Chromatin immunoprecipitation (ChIP)

The ChIP assay was performed as described previously [60] with some minor changes described below. In brief, 1 × 10^6^ cells were seeded and then, after 24 hours were fixed for 15 min. at room temperature in 1× formaldehyde solution (HCOH 1%, NaCl 10 mM, EDTA 0.1 mM, EGTA 0.05 mM, Hepes 5 mM in L-15 cell medium). After cell lysis in buffer L1 (Tris-HCl 50 mM pH 8.0, EDTA 0.2 mM pH 8.0, NP40 0.1%, Glycerol 10% including a 1× cocktail of protease and phosphatase inhibitors (Sigma Aldrich)), the nuclei were recovered by centrifugation (5 min. at 4°C at 3000 rpm) and resuspended in lysis buffer L2 (Tris-HCl 50 mM pH 8.0, EDTA 5 mM, SDS 1%). The sonicated chromatin (8 × 5s pulses) solution was diluted to a final volume of 3 ml with DB buffer (Tris-HCl 50 mM pH 8.0, EDTA 5 mM, NaCl 200 mM, NP40 0.5%). For each test (α-YB-1, no antibody (Ab) and α-IgG controls), 1 ml of chromatin solution was used. Immunoprecipitation was performed at 4°C over-night on a rotating platform. The antibody concentration was chosen according to the manufacturer's instructions. To recover the immunocomplexes (antibody plus chromatin) we used magnetic beads (Dyna beads Protein G, ref. 1004D, Novex Life-Technology). After extensive washing, bound DNA fragments were eluted and subsequently analyzed by PCR. PCR primers are reported in Supplementary Table S1. The 150 bp or 450 bp PCR products were visualized by 2% Agarose Gel Electrophoresis.

### Statistical analysis

CircWaveBatch 3.3 (by courtesy of Dr. Roelof Hut, http://www.euclock.org/component/zoo/item/deliverables.html) was used to identify sinusoidal differential expression during the 24-hour cycle. This statistical algorithm tests each expression profile against a harmonic curve by varying the phase and period length. A F-test was used to measure the goodness-of-fit and the transcript was considered rhythmic when the harmonic regression was statistically significant (*p* < 0.05). Results of the CircWave analysis are represented in Supplementary Table S1. One-way analysis of variance (ANOVA) followed by Bonferroni's multiple comparison tests was also performed using GraphPad Prism 4.0 ( http://www.graphpad.com).

Statistical analysis of the real time bioluminescence data from the cyclin A2 luciferase reporter construct was performed using the MATLAB R2015b software as follows: Each night/day cycle was processed separately. A regression routine of order one delivered a mean slope per cycle. Equal cycles were averaged per well. To obtain cycle changes between night and day, the mean slope of all cycles was subtracted. A *t*-test for differences was analysed (see Supplementary Table S1).

All the statistical results are expressed as means +/– SD of three biological replicates where for each *in vivo* experiment a minimum of *n* = 3 fish were used per timepoint. In the statistical tests *p* < 0.05 was considered statistically significant. In each figure, *p* < 0.05, *p* < 0.001 and *p* < 0.0001 are represented by *, ** and *** respectively.

## SUPPLEMENTARY MATERIALS FIGURES AND TABLES


