# Supplementary material for: The tumor-associated YB-1 protein: new player in the circadian control of cell proliferation
Source: Oncotarget. 2016 Dec 20;8(4):6193–205. doi: 10.18632/oncotarget.14051 (PMC5351623; doi:10.18632/oncotarget.14051)
Supplement: Supplementary file 1 [file oncotarget-08-6193-s001.pdf]

The tumor-associated YB-1 protein: new player in the circadian control of cell proliferation

Supplementary Materials

Supplementary Table S1: Statistical analysis

name	Figure	pAnova Circ-wave	*T*-Test/Anova/Bonferroni
Fin-YB-1 N-ter nuclear LD (IMF)	1A,B	-	$P < 0.0001$ (*T*-Test Microsoft Excel)
Fin-YB-1 N-ter nuclear LD first day (WB)	2A	$P = 0.0001$	$P < 0.0001$
Fin-YB-1 N-ter nuclear LD second day WB	2A	$P = 0.0277$	$P < 0.001$
Fin-YB-1 Full nuclear LD first day (WB)	2B	$P < 0.00001$	$P < 0.0001$
Fin-YB-1 Full nuclear LD second day (WB)	2B	$P = 0.0005$	$P < 0.001$
Fin-YB-1 N-ter nuclear DD (WB)	2C	$P < 0.00001$	$P < 0.001$
PAC-2 cells YB-1 N-ter LD (IMF)	3A	$P < 0.00001$	$P < 0.0001$
PAC-2 cells YB-1 N-ter nuclear LD (WB)	3B	$P < 0.00001$	$P < 0.001$
CLOCK1 DN cells YB-1 N-ter LD (IMF)	3C	ns $P = 0.061$	ns $P > 0.05$
CLOCK1 DN cells YB-1 N-ter LD (WB)	3D	ns $P = 0.0617$	ns $P > 0.05$
h per2 DEX in HEK 293 cells (qRT-PCR)	4A	$P = 0.03822$	
h bmal1 DEX in HEK 293 cells (qRT-PCR)	4B	$P = 0.04847$	
H YB-1 DEX in HEK 293 cells (WB)	4D	$P = 0.02372$	
Ubc9b (ube2ib) LD (qRT-PCR)	5C	$P = 0.0004$	
Ubc9b (ube2ib) DD (qRT-PCR)	5C	$P = 0.0282$	
PAC-2 cells *zf cyclinA2 pr.-Luc LD*	6A	-	$P = 0.004$ (*T*-Test MATLAB)
CLOCK1 DN cells *zf cyclinA2 pr.-Luc LD*	6A	-	ns $P = 0.6$ (*T*-Test MATLAB)
Fin-YB-1 F. nuclear DD (WB)	S2D	$P < 0.0001$	$P < 0.05$
zf Yb1 LD (qRT-PCR)	S4A	ns $P = 0.8052$	ns $P > 0.05$
zf Yb1 DD (qRT-PCR)	S4A	ns $P = 0.066$	$P < 0.004$ ct 0 vs ct 21
zf Per1b LD (qRT-PCR)	S4B	$P < 0.00001$	$P < 0.0001$
zf Per1b DD (qRT-PCR)	S4B	$P < 0.00001$	$P < 0.0001$
zf cyclinA2 LD (qRT-PCR)	S4C	$P < 0.00001$	$P < 0.001$
zf cyclinA2 DD (qRT-PCR)	S4C	$P < 0.0001$	$P < 0.001$
PAC-2 cells YB-1 N-ter nuclear DD, no serum (WB)	S5F	$P = 0.015$	$P < 0.05$
zf ubc9a (ube2ia) LD (qRT-PCR)	S6A	$P < 0.00001$	
zf ubc9a (ube2ia) DD (qRT-PCR)	S6A	$P < 0.00001$	
zf sumo1 LD (qRT-PCR)	S6B	$P = 0.0002$	
zf sumo1 DD (qRT-PCR)	S6B	$P = 0.0033$	
zf pias LD (qRT-PCR)	S6C	$P < 0.00001$	
zf pias DD (qRT-PCR)	S6C	ns $P = 0.2$	
zf sae1 LD (qRT-PCR)	S6D	$P = 0.0016$	
zf sae1 DD (qRT-PCR)	S6D	$P = 0.008$	
zf cyclinA2 LD cells (qRT-PCR)	S7B	$P < 0.00001$	$P < 0.001$

Supplementary Table S2: PCR primers for qRT-PCR and ChIP assays

zf β -actin	F: GCCTGACGGACAGGTCAT	R: ACCGCAAGATTCCATACCC
zf Yb-1	F: TACCCACCATACTTCGTGCG	R: GCGGTAGTTGAAGTTGCGAC
zf per1b	F: CCGTCAGTTTCGCTTTTCTC	R: ATGTGCAGGCTGTAGATCCC
zf *cyclin A2 (ccna2)*	F: TAGATTGCGATCCCTTCCTC	R: CCTGTTGAGCGTGTTGAGAA
Zf ubc9b (ube2ib)	F: CAGGATCCAGCTCAGGCAGA	R: CGACGGGGAGAACTTTTTGG
Zf ubc9a (ube2ib)	F: AGGCCGAGGCATACACGATT	R: GTGGTGGACGGGGTGAGGAG
zf sae1	F: GCCAAAGATCGACCCCAATG	R: TGCGCTTCAGGCTGCTTTTA
zf PIAS1	F: TCCGTCACTGTCCAGCCTCT	R: ACCTGAGGCATGCTGGGAGT
zf sumo1	F: AGAGACAGGGTGTACCCGTGAA	R: TGGGGGTCAGGTTGTCTGTG
zf *cyclin A2 prom. 150 bp*	F: AACCCGCCACCACAAGTTTATT	R: GGCAACGTCATCAGGGGTAGA
zf *cyclin A2 prom. 450 bp*	F: AACCCGCCACCACAAGTTTATT	R: GCACGCTAACCAGAGAAAGC
h - gapdh	F: ACGTGTCAGTGGTGGACCTGA	R: AGCGTCAAAGGTGGAGGAGTG
h per2	F: CCACGAGAATGAAATCCGCT	R: CCCGCACCTTGACCAGG
h bmal1	F: GAAATCATGGAAATCCACAGGATAA	R: GAGGCGTACTCGTGATGTTCAAT

Supplementary Table S3: siRNAs

iBONI zfYB-1 siRNA	5′-3′ Guide<br>UUCUCCUUAUCCUCCUCUCCCCC	5′-3 Passenger<br>GGGGGAGAGGAGGAUAAGGAGAA
iBONi siRNA Negative Control 1	5′-3′ Guide<br>UUGUACUACACAAAAGUACCCCC	5′-3 Passenger<br>GGGGGUACUUUUGUGUAGUACAA
iBONi siRNA Negative Control 2	5′-3′ Guide<br>GAACGAAUUUAUAAGUGGCCCCC	5′-3 Passenger<br>GGGGGCCACUUAUAAAUUCGUUC
iBONi siRNA Negative Control 3	5′-3′ Guide<br>UUGUACUACACAAAAGUACCCCC	5′-3 Passenger<br>GGGGGUACUUUUGUGUAGUACAA

Supplementary Table S4: Primary and secondary antibodies

Abcam ab12148 α-YB-1	Residues 1-100 of human YBX-1 (N-ter)
Santa Cruz sc-101198 α-YB-1 (59 Q)	Against recombinant full α -human YBX-1 (F)
Sigma-Aldrich α-YB-1 (C-ter) Y0396	Residues 307-324 of human α –YBX-1 (C-ter)
Santa Cruz Sc- 5308 α-SUMO-1 (D-11)	Residues 1-101 of human–SUMO1
Sigma-Aldrich V9131 α-vinculin	α –vinculin monoclonal clone hVIN-1
Cell Signaling α-H3 (9715S)	α-H3
Cell Signaling α-PH3 (9701S)	α-PH3
Rabbit Polyclonal, Goat, HRP	Cell Signaling (7074S)
α-Mouse Monoclonal,Goat,HRP (7076S)	Cell Signaling (7076S)
α-Mouse Alexa fluor 546 F(ab')2	Life Technologies (A11018)
α-Rabbit Alexa fluor 546 F(ab')2	Life Technologies (A11017)

Supplementary Table S5: Cloning details

Name	Vector	Insert	Details
zf YB-1 cDNA	pGEM-T easy (Promega)	PCR	primer in 5′ UTR F: gtggagagatgtgacagaatatcg<br>primer in 3′ UTR R: gtttttatttcagcttgccttttg
zf YB-1-GFP(3′)	pcDNA6-V5/HisB GFP<br>Afl2 –EcoR1	PCR	primer F: ccgg**cttaag**caacaagaaaacaacATG<br>primer R: gcggt**TTGaattc**gctccgccctgttc<br>Stop codon TAA mutated to CAA
5xMyc-zfYB-1	pCS2 MTK 5xMyc<br>(Invitrogen)<br>EcoR1- Xho1	PCR	ATG mutated to TTC:<br>primer F: gaaaa**gaaTTC**agcagcgaggccgagaca<br>primer R: ggatggt**ctcgag**gtttaagccggtggcggt
zf cyclin A2 prom.	pGEM-T easy (Promega)	PCR	primer F: ccgccaccacaagtttattc<br>primer R: gcacgctaaccagagaaagc
zf cyclin A2-luc reporter	pGL3 basic<br>(Promega) Sac1-Nco1		SacI-NcoI from<br>zf cyclin A2 prom. pGEM-T easy

YB-1 sequences alignment

```
                                                                                             Cold-shock domain
                     *********                                   **   * *************************************************
H.sapiens          1 MSSEAETQQPPAAPPAAPALSAADTKPGTTGSGAGSGGPGGLTSAAPAGGDKKVIATKVLGTVKWFNVRNGYGFINRNDTKEDVFVHQTAIKKNNPRKYL   100
M.musculus         1 MSSEAETQQPPAAP--AAALSAADTKPGSTGSGAGSGGPGGLTSAAPAGGDKKVIATKVLGTVKWFNVRNGYGFINRNDTKEDVFVHQTAIKKNNPRKYL   98
D.rerio            1 MSSEAETQQPPQPA-------------------ADAESPSSPAAAATA-GDKKVIATKVLGTVKWFNVRNGYGFINRNDTKEDVFVHQTAIKKNNPRKYL   80

                     ******************************************* ******** *** ** ***     *  ** ** ******    *****    **
H.sapiens        101 RSVGDGETVEFDVVEGEKGAEAANVTGPGGVPVQGSKYAADRNHYRRYPRRRGPPRNYQQNYQNS---ESGEKNEGSESAPEGQ--AQQRRPY--RRRRF   193
M.musculus        99 RSVGDGETVEFDVVEGEKGAEAANVTGPGGVPVQGSKYAADRNHYRRYPRRRGPPRNYQQNYQNS---ESGEKNEGSESAPEGQ--AQQRRPY--RRRRF   191
D.rerio           81 RSVGDGETVEFDVVEGEKGAEAANVTGPGGVPVQGSKYAADRNRYRRYPRRRAPPRDYQENYQSDPEAEPREKREGAESAPEGEMQQQQRRPTYPGRRRY   180

                    ***  ** * *** * *    **  **       *  **   *  *  *** **  ***** *     * ********   * * *  *************
H.sapiens        194 PPYYMRRPYGRRPQYSNPPVQGEVMEGADNQGA--G-EQG-RPVRQNMYRGYRPRFRRGPPRQRQPREDGNEEDKENQGDETQGQQPPQRRYRRNFNYRRR   290
M.musculus       192 PPYYMRRPYARRPQYSNPPVQGEVMEGADNQGA--G-EQG-RPVRQNMYRGYRPRFRRGPPRQRQPREDGNEEDKENQGDETQGQQPPQRRYRRNFNYRRR   288
D.rerio          181 PPYFVRRRYGRRPPYTNSQ-RGEMTEGGEGEENQGGPDQGNKPMRQNYYRGFRP--SRGPPRPRPVR-DG-EEDKENQSESGQNQEPRQRRYRRNFNYRRR   276

                     *    ******  **    *    *********
H.sapiens        291 RPENPKPQDGKETKAADPPAENSSAPEAEQGGAE   324
M.musculus       289 RPENPKPQDGKETKAADPPAENSSAPEAEQGGAE   322
D.rerio          277 RPQTTKPQDGKDSKAADASADKSAAPEAEQGGAD   309
```

Supplementary Figure S1: Human, mouse and zebrafish YB-1 protein alignment. Multiple alignments of YB-1 protein sequences derived from H. sapiens (NP_004550), M. musculus (NP_035862) and zebrafish (D. rerio NP_001119929.1). Asterisks (*) indicate conserved amino acids (aa) between the three species. The Cold-Shock domain (CSD) is indicated in red. Black bordered squares indicate the three putative sumoylation sites predicted by GPS-SUMO 1.0 software. The sites are defined as consensus ψKxE/D or inverted E/DxKψ where ψ = hydrophobic aa; x = any aa. In zfYB-1: DSKA 287-290 aa is a predicted canonical inverted sumoylation site. Instead, TKED 60-63 aa and EKRE 151-154 aa are scored as non-canonical sumoylation sites due to the absence of a ψ amino acid.

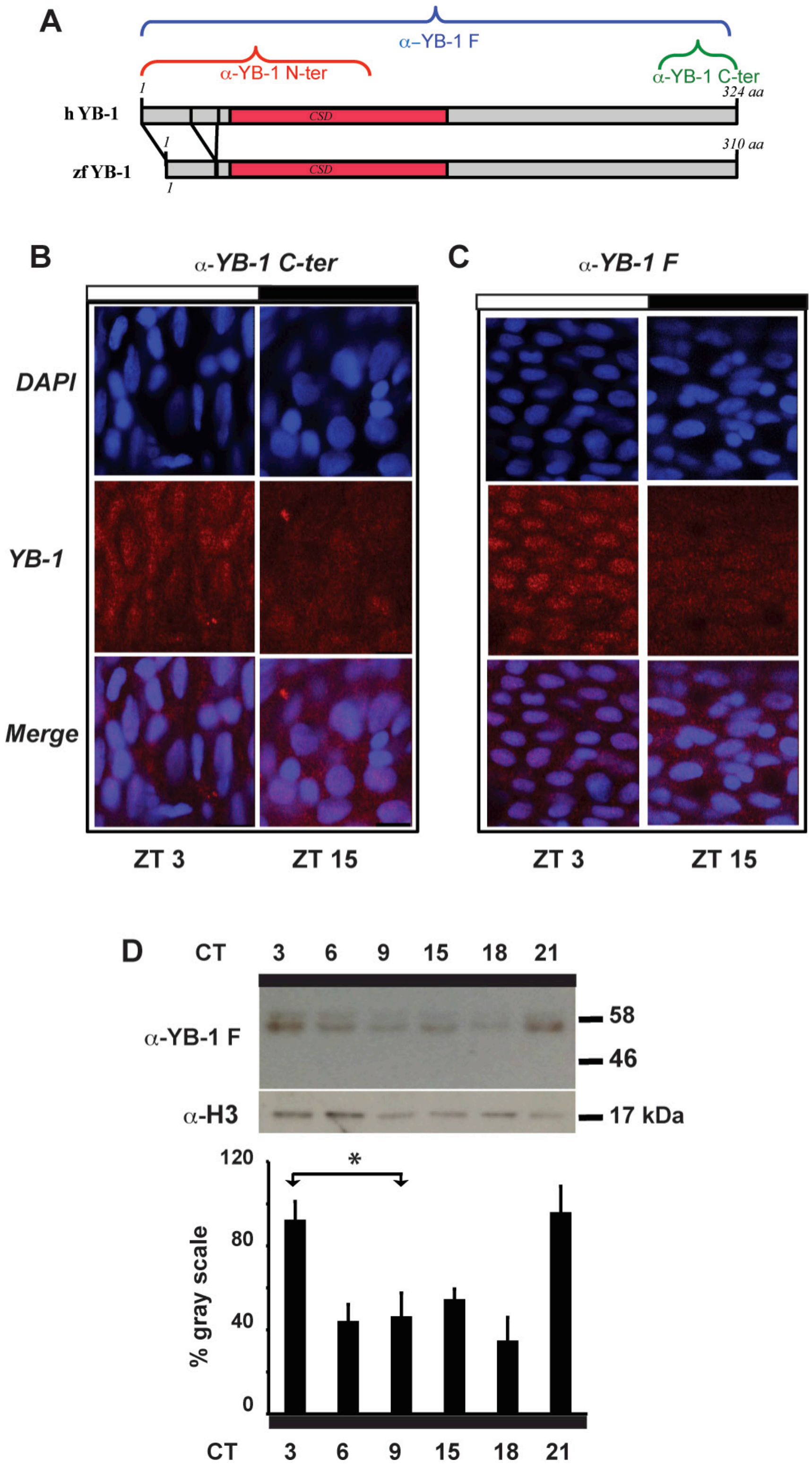

Supplementary Figure S2: zfYB-1 protein expression in caudal fins. (**A**) Schematic representation of the human and zebrafish YB-1 proteins. Cold Shock Domains (red bars, CSD) and the portions recognized by the 3 antibodies, α-YB-1 N-ter, C-ter and F are indicated. (**B**, **C**) Immunofluorescence analysis of zfYB-1 protein in the caudal fin at ZT3 (light phase) and ZT15 (dark phase) using the α-YB-1 C-ter (B) and α-YB-1 F antibodies (C). Panel also shows DAPI staining and Merge, which combines both the DAPI and YB-1 signals. White and black bars above the panel indicate the corresponding lighting conditions. (**D**) Western blot analysis of zfYB-1 levels in fin nuclear extracts prepared during the second day in DD following transfer from LD cycle conditions. α−H3 was used for normalization. Quantification of western blot signals in panel D, was expressed as % of gray scale relative to the highest peak time point and plotted on the y-axes. CT times for each sample are plotted on the x-axis. Statistical significance is indicated above the graph by asterisks (*). White and black bars indicating the corresponding lighting conditions as well as the ZT (for LD) and CT (for DD) times are indicated above each panel.

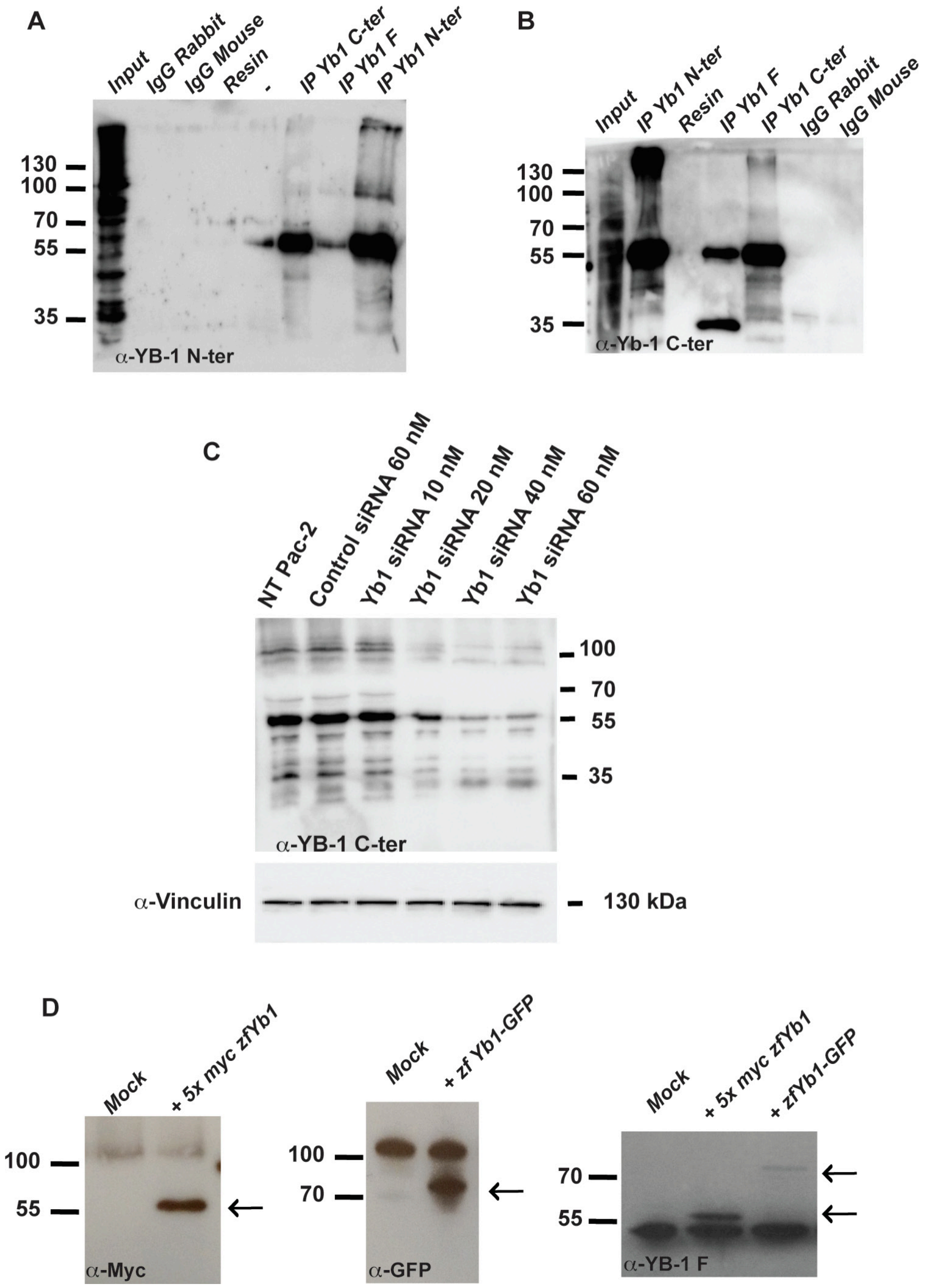

Supplementary Figure S3: Immunoprecipitation (IP), silencing (siRNA) and overexpresion of of zfYB-1. (**A**) Western blot analysis using α-YB1 N-ter and (**B**) α-YB1 C-ter antibodies of IPs prepared from cell total extracts using each of the three YB-1 antibodies. (**C**) Western blot analysis using α-YB1 C-ter of total cell extracts prepared from Pac-2 cells transfected with increasing concentrations of zfYB-1 siRNA and normalized using an α-vinculin antibody. As a control, Pac-2 cells were transfected with a high dose (60 nM) of a pool of scrambled siRNAs (control). (**D**) Control western blot analysis using α−Myc, α−GFP and α-YB-1 F antibodies of total cell extracts prepared from Pac-2 cells transfected with 5xMyc-zfYB-1 or zfYB-1-GFP expression constructs for zfYB-1.

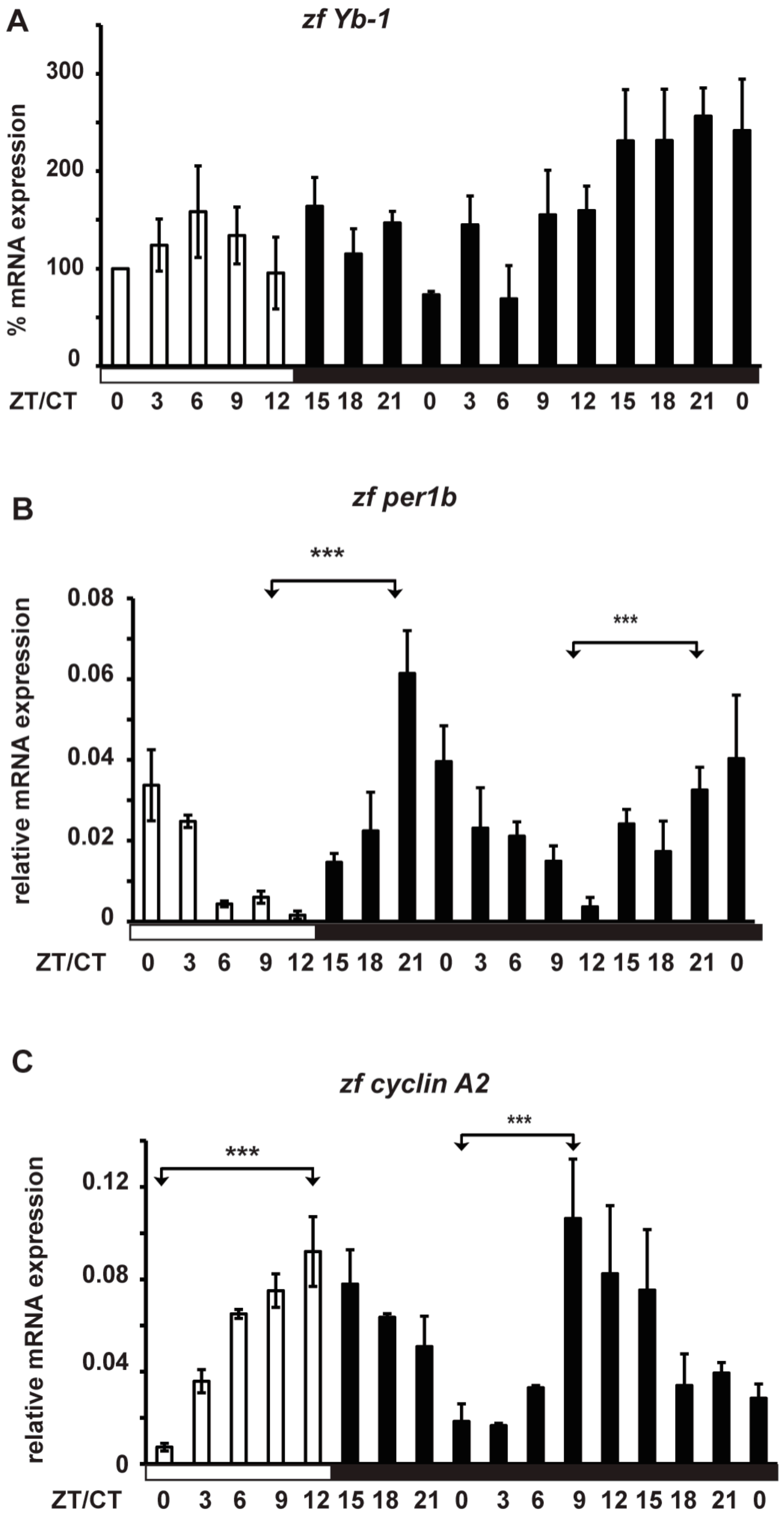

Supplementary Figure S4: Real time qRT-PCR analysis of zebrafish caudal fin gene expression. qRT-PCR analysis of *zfYB-1* (**A**) *zfper1b* (**B**) and *zfcyclin A2* expression (**C**) during exposure for 24 hours to LD cycle conditions followed by 24 hours in DD. RNA samples were harvested at 3 hrs intervals and relative mRNA levels calculated and plotted on the y-axes. ZT and CT times are plotted on the x-axes. Relative expression of *β-actin* mRNA was used for normalization. Statistical significance is indicated above the graph by asterisks (*).

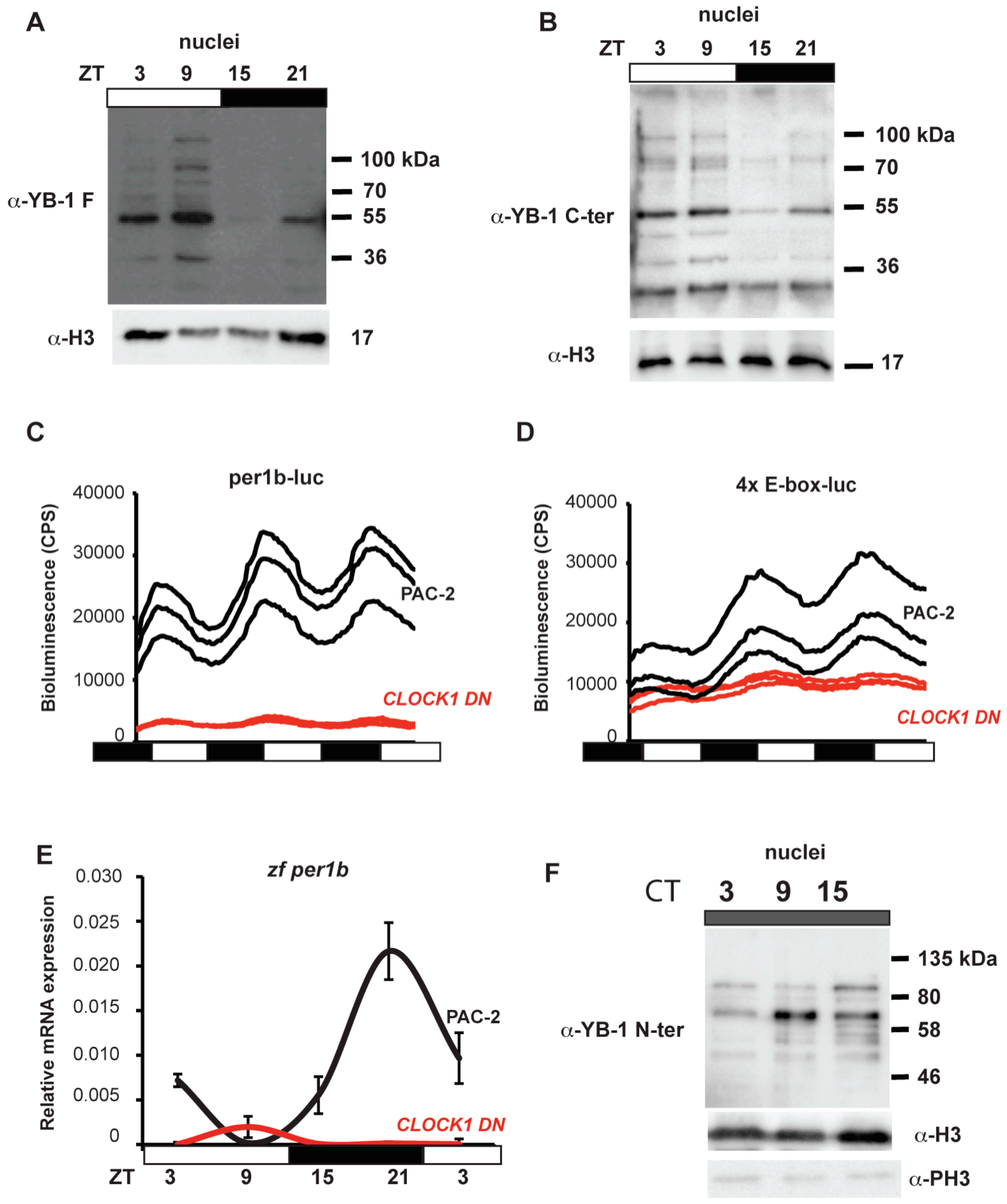

Supplementary Figure S5: Nuclear YB-1 expression in PAC-2 and CLOCK1 DN cells. (**A**, **B**) Western blot analysis of nuclear extracts from PAC-2 cells using (A) the α-YB-1 F and (B) α-YB-1 C-ter antibodies. Histone H3 was used for normalization. (**C**, **D**) Real time bioluminescence assay of PAC-2 (black traces) and CLOCK1 DN (red traces) cells under LD cycles transfected with (C) the *per1b-luc* and (D) the *4x E-box–luc* reporters. Three independent assays for each cell line are represented. Bioluminescence levels (counts per seconds, CPS) are plotted on the y-axis. White and black bars indicate the corresponding lighting conditions. (**E**) qRT-PCR analysis of *zf per1b* in PAC-2 (black traces) and CLOCK1 DN (red traces) cells exposed to a LD cycle. RNA samples were harvested at 6 hrs intervals for 30 hours and the calculated, relative mRNA levels are plotted on the y-axes. ZT times are plotted on the x-axes. Relative expression of *β-actin* mRNA was used for normalization. (**F**) Western blot analysis for YB-1 in nuclear extracts prepared from confluent PAC-2 cells maintained in LD followed by 48 hours in constant darkness in the absence of serum before sampling. The α-YB-1 N-ter antibody was used. Histone H3 was used as an internal loading control and P-H3 was used as a control for lack of proliferation. White and black bars indicating the corresponding lighting conditions as well as the ZT or CT times are represented in each panel.

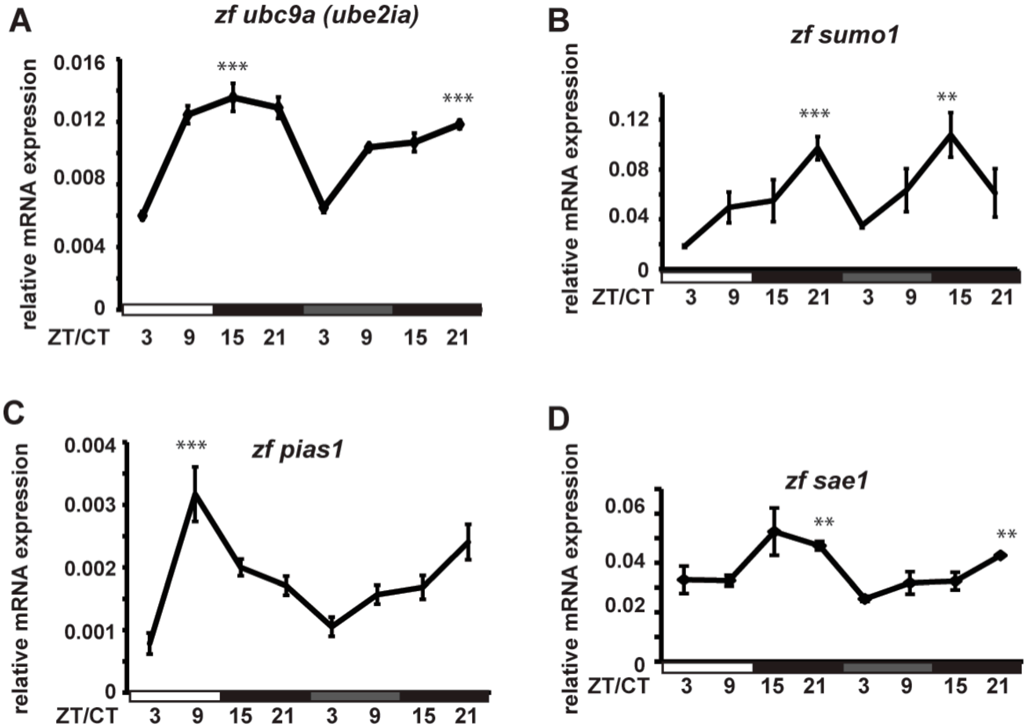

Supplementary Figure S6: Real time qRT-PCR analysis of zebrafish genes involved in SUMOylation. qRT-PCR analysis of *zf ubc9a* NM_131833.2 (**A**), *zf sumo1* NM_213159.1 (**B**), *zf pias1* XM_687829.4 (**C**) and *zf sae1* NM_001002058.1 (**D**) during exposure for 24 hours to LD cycle followed by 24 hours in DD. RNA samples were harvested at 6 hrs intervals and relative mRNA levels calculated and plotted on the y-axes. ZT and CT times are plotted on the x-axes. Relative expression of *β-actin* mRNA was used for normalization. Statistical significance between peak and trough points is indicated above the graph by asterisks (*). Results of the CircWave analysis are represented in Supplementary Table S1.

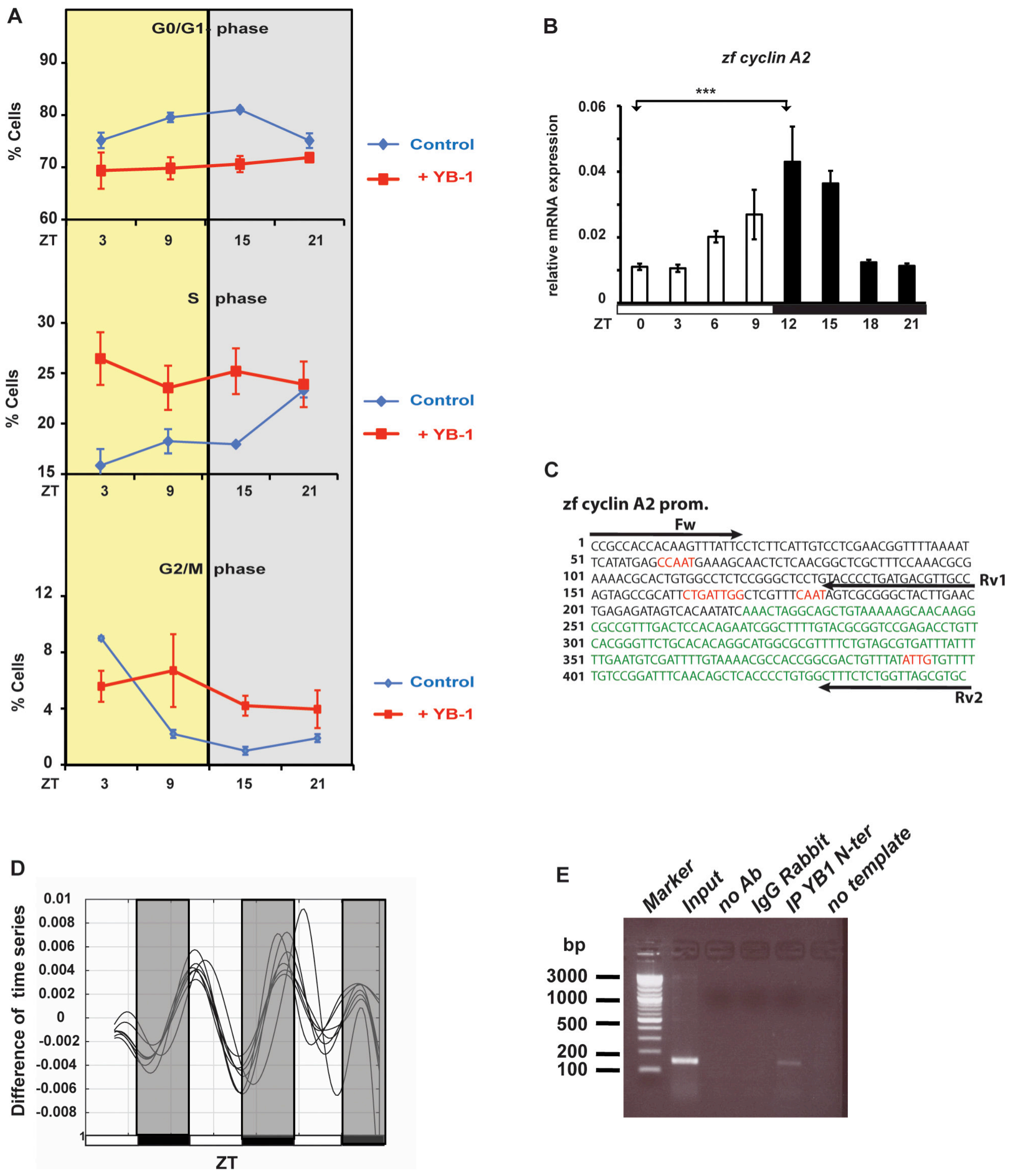

Supplementary Figure S7: Regulation of *zfcyclin* A2 expression by YB-1. (**A**) DNA cell cycle profile of PAC2 cells in the absence (blue traces) or presence of a transfected YB-1 expressing construct (red traces) during a 24 hours LD cycle. DNA cell cycle distribution, G0/G1 (upper panel), S (central panel) and G2M (lower panel), was determined by PI staining and FACS flow cytometry. DNA histograms were analyzed by BD accuri C6 analysis software. Samples were harvested at 6 hrs intervals and the percentages of cells in the specific cell cycle phases are plotted on the y-axis. ZT times are plotted on the x-axis. (**B**) qRT-PCR analysis of *zfcyclin A2* expression during 24 hours under LD cycle conditions in PAC-2 cells. Samples were harvested at 3 hrs intervals and relative mRNA levels were plotted on the y-axis. ZT times are plotted on the x-axis. *β-actin* expression was used for normalization. Statistical significance is indicated above the graph by asterisks (*). (**C**) Sequence of the *zfcyclin A2* promoter region cloned upstream of a luciferase reporter gene. In red are indicated the putative forward and reverse CCAAT binding sites for YB-1. Black arrows indicate the forward (Fw) and the two reverse primers (Rv1 and Rv2) used for the ChIP assay. The sequence in green indicates the 5`UTR region. (**D**) Graphical representation of the real time bioluminescence assay results from PAC-2 cells transfected with the *cyclin A2 pr.* luciferase reporter. The difference of filtered time series bioluminescence is plotted on the y-axis and the ZT times on the x-axis. Results are plotted as 8 independent replicates (see materials and methods for details of the calculations and Figure 6A for raw data). White and black bars indicate the corresponding lighting conditions. (**E**) Agarose gel showing the amplified products from ChIP assays. DNA fragments of 150 bp were amplified using the primers shown in Supplementary Table S2. Input and negative controls are indicated.
